# Sub-10 nm PdNi@PtNi Core–Shell Nanoalloys for Efficient Ethanol Electro-Oxidation

**DOI:** 10.3390/molecules29204853

**Published:** 2024-10-13

**Authors:** Qian Su, Lei Yu

**Affiliations:** College of Chemistry & Chemical and Environmental Engineering, Weifang University, Weifang 261061, China; 20220033@wfu.edu.cn

**Keywords:** core–shell structure, ternary alloys, fuel cells, synergistic effect

## Abstract

By controlling the structure and composition of Pt-based nanoalloys, the ethanol oxidation reaction (EOR) performances of Pt alloy catalysts can be effectively improved. Herein, we successfully synthesis sub-10 nm PdNi@PtNi nanoparticles (PdNi@PtNi NPs) with a core–shell structure by a one-pot method. The sub 10 nm core–shell nanoparticles possess more effective atoms and exhibit a synergistic effect which can lead to a shift in the d-band center and alter binding energies toward adsorbates. Due to the synergistic effect and unique core–shell structure, the PdNi@PtNi NP catalysts exhibit excellent electrocatalytic performance for ethanol oxidation reactions in alkaline, achieving 9.30 times more mass activity and 7.05 times more specific activity that of the state-of-the-art Pt/C catalysts. Moreover, the stability of PdNi@PtNi NPs was also greatly improved over PtNi nanoparticles, PtPd nanoparticles, and commercial Pt/C. This strategy provides a new idea for improving the electrocatalytic performance of Pt-based catalysts for EORs.

## 1. Introduction

In recent decades, the rising demand for energy and the growing concern over environmental issues have made the development of renewable fuel cells increasingly important [1,2,3,4,5,6,7]. Direct ethanol fuel cells (DEFCs) have garnered growing interest due to their high energy conversion efficiency, as well as the convenience of ethanol storage and transportation [8,9]. Currently, Pt is widely used as an electrocatalyst at the anode for alcohol oxidation reactions (EORs) [10,11,12,13]. However, the high cost of Pt-based catalysts and their vulnerability to poisoning by surface-adsorbed CO_ads_ have posed significant challenges to their large-scale commercial application in EORs [8,11,14]. Therefore, improving the utilization and catalytic performance of Pt catalysts remains an urgent issue that needs to be addressed.

For EORs, two parallel but competing electrochemical pathways exist, namely the complete C1 pathway and the incomplete C2 pathway [11]. The C1 pathway involves the cleavage of the high-energy C–C bond, leading to the complete electro-oxidation of ethanol into more desirable products in an alkaline solution. In contrast, the C2 pathway forms acetic acid through a four-electron exchange process. Notably, the C1 pathway, which transfers 12 electrons and releases greater electrical energy, is more electrochemically favorable for DEFCs. However, Pt catalysts are easily poisoned by the intermediate species (CO_ads_) produced by the reaction, leading to the decrease in activity. And the oxidation removal of CO-based intermediates (CH_3_CO_ads_ and CO_ads_) by adsorbed OH (OH_ads_) is of critical importance in promoting EOR performance [11]. According to the literature, alloying with other 3d transition metals is beneficial for electronic structure optimization. The synergistic effect between metals (possessing different d-band centers) allows for an electronic charge transfer between them after alloying noble metals with transition metals, demonstrating a useful method to resist CO poisoning and enhancing the catalytic performance of the catalysts, such as PtMo [15], PtFe [2], PtRh [13] PtCuMn [16], PtCuFe [17], and PtPdSn [18]. Gao’s group [16] reported a dendritic defect-rich PdCuFe ternary nanoalloy and showed an excellent activity and durability for electro-oxidation because of the synergistic effect. Furthermore, specific surface structures can be constructed to provide a larger specific surface area and more defect sites, which can effectively enhance the electrocatalytic performance [9,19,20,21,22,23,24]. Shuiping Luo et al. [24] synthesized a PtBi@PtRh core–shell by atomic galvanic replacement and electrochemical dealloying. Benefiting from the Rh-tailored Pt (110) surface with tensile strain, the PtBi@PtRh nanoplates exhibited record-high and all-round superior electrocatalytic performance for ethanol oxidation electrolytes. The core–shell structure of Pt-based nanocrystals exhibited superior catalytic activity in electro-oxidation reactions, primarily because of the interaction between the Pt shell and the core. It is known that the oxidation removal of CO-based intermediates (CH_3_CO_ads_ and CO_ads_) by adsorbed OH (OH_ads_) is of critical importance in promoting electrochemical performance [11]. The core–shell structure optimizes the binding energetics of intermediates on the Pt surface by adjusting the surface Pt strain [25,26,27,28]. In addition, the core–shell structure can significantly provide more effective atoms and reduce the cost of Pt [29]. Therefore, constructing specific surface structures for Pt-based ternary alloy catalysts will be an ideal choice for electrocatalysts.

Currently, Pt-based core–shell nanoalloys have been already reported [30,31,32,33]. However, the majority of these nano core–shell structures are produced using multi-step synthesis methods or on a large-scale [34,35,36,37,38]. Kuttiyiel et al. [39] developed an approach of forming Pt/Pd/C electrocatalysts by the galvanic displacement of an underpotentially deposited method. A Cu monolayer is formed on the surface of a Pd nanoparticle using the underpotentially deposited method. Then, the Pt monolayer is deposited by the galvanic displacement of the Cu to obtain the core–shell structure. Thus, developing a simple and environmentally friendly synthesis method to prepare small size Pt-based core–shell structures is of great significance.

In this work, we strategically designed sub-10 nm PdNi@PtNi nanoparticles (PdNi@PtNi NPs) with a core–shell structure as efficient electrocatalysts for EORs in alkaline media. The PdNi@PtNi NPs were prepared though a one-pot solvothermal method. Through downsizing the particle sizes of catalysts, the sub-10 nm PdNi@PtNi not only reduces the usage of noble metals but also processwa more active sites to be available for the adsorption and reaction of ethanol and its intermediates. Furthermore, the PtNi shell and the PdNi core in PdNi@PtNi NPs are beneficial for CO anti-poisoning during EORs, thus improving the overall catalytic activity. Specifically, the PdNi@PtNi NPs demonstrated superior electrocatalytic performance for EORs, achieving mass and specific activities of 9.30 and 7.05 times higher, respectively, than those of the state-of-the-art Pt/C catalysts. CO stripping results further show that CO is more easily oxidized on PdNi@PtNi NPs compared to PtNi NPs, PtPd NPs, and Pt/C. This enhanced CO oxidation helps mitigate the CO poisoning of Pt, thereby improving the electrocatalytic performance for alcohol oxidation.

## 2. Results and Discussion

**Figure 1:**
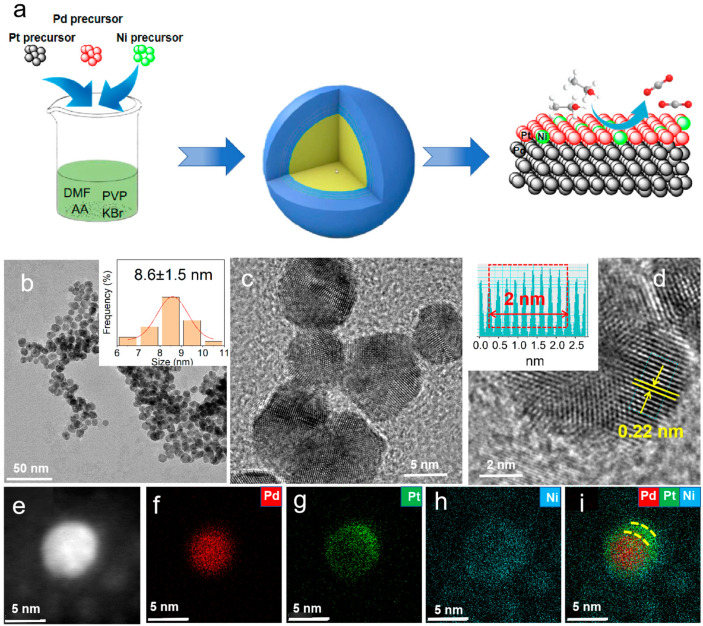
(**a**) Illustrations of the synthesis of PdNi@PtNi NPs. (**b**–**d**) HR-TEM images of PdNi@PtNi NPs. Inset in b shows the diameter distribution of individual nanoparticles in PdNi@PtNi NPs. (**e**–**i**) HAADF-STEM-EDS mapping images of PdNi@PtNi NPs.

As shown in Figure 1a, in the synthesis process, platinum acetylacetonate, palladium acetylacetonate, and nickel acetylacetonate were selected as metal precursors. AA was used as the reducing agent, KBr as the structure-directing agent, PVP as the stabilizer, and DMF as the solvent. The core–shell structured PdNi@NiPt NPs were obtained using a solvothermal method. As a contrast, the PtNi nanoparticles (Figure S1) and PtPd nanoparticles (Figure S2) were also prepared via a similar solvothermal method.

Figure 1b presents the TEM images of the PdNi@PtNi sample. As shown in the images, the majority of the products are small nanoparticles with a high yield approaching 95% and an average edge length of around 8.6 nm. HRTEM characterization was also conducted to examine the surface structure and external atomic arrangement of the catalysts. Figure 1c,d show that the lattice spacing of PdNi@PtNi is 0.22 nm, matching the {111} crystal plane of face-centered cubic (fcc) Pt. To gain a deeper understanding of the elemental distribution and structural composition of PdNi@PtNi, EDS mapping was carried out. As shown in the results (Figure 1f–i), Pd is mainly distributed at the center core (red), while Pt elements comprise the shell region (green and blue). Meanwhile, the Ni element is uniformly distributed throughout the whole nanoparticle. The results present the formation of the PdNi@PtNi core–shell structure with PdNi as the core and PtNi as the shell, which is confirmed by the XPS results in the following discussiond. The molar ratios of Pt/Ni/Pd in the PdNi@PtNi alloys, as measured by ICP-OES, are 10.1/29.8/60.1, respectively (Table S1).

**Figure 2:**
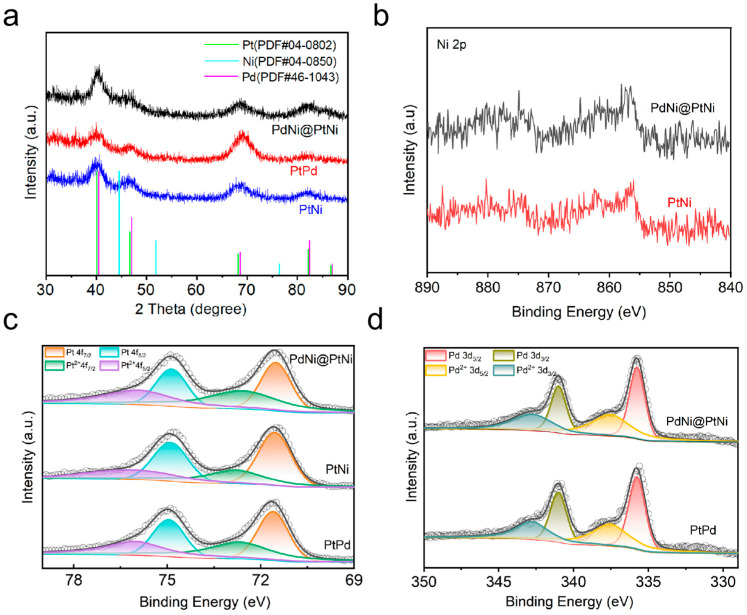
(**a**) XRD patterns of PdNi@PtNi NPs, PtNi NPs, and PtPd NPs. (**b**) Ni 2p XPS spectra of the catalysts. (**c**) Pt 3d XPS spectra of the catalysts. (**d**) Pd 4f XPS spectra of the catalysts.

To obtain an in-depth understanding of the composition and structure of PdNi@PtNi NP, PtNi NP, and PtPd NP catalysts, X-ray diffraction (XRD) and X-ray photoelectron spectroscopy (XPS) measurements were performed (Figure 2 and Figure S3). Figure 2 presents the XRD (Figure 2a) and XPS (Figure 2b–d) spectra of the PdNi@PtNi NPs, PtNi NPs, and PtPd NPs. As shown in Figure 2a, the four diffraction peaks of PdNi@PtNi NPs corresponding to (111), (200), (220), and (311) are positioned between the peak locations of pure Pt (PDF#04-0802), Pd (PDF#46-1043), and Ni (PDF#04-0850) in their respective XRD patterns. This shift is primarily due to the substitution of some Pt atoms by Ni and Pd atoms, leading to a contraction of the Pt lattice and causing the peak positions to shift toward those of Ni and Pd, thereby confirming the formation of the PdNi@PtNi alloy. This result is consistent with the HRTEM and EDS analysis findings. According to the literature [2,18,21,40,41,42,43], the electronic interactions between different metals can cause shifts in the d-band center relative to the Fermi level. A downward shift of the d-band center can reduce adsorption energy, while an upward shift can enhance adsorption energy. As shown in Figure 2c, peaks in PdNi@PtNi NPs at 74.86 eV and 71.51 eV are assigned to the metallic Pt 4f_5/2_ and Pt 4f_7/2_ peaks, respectively. And the peaks at 76.02 eV (Pt 4f_5/2_) and 72.67 eV (Pt 4f_7/2_) are attributed to Pt^2+^. Similarly, the Pt 4f peaks at 74.91 and 71.59 eV in PtPd nanoparticles (NPs) and at 74.9 and 71.55 eV in PtNi NPs correspond to the metallic state of the Pt species. The Pt 4f peaks observed at 76.23 and 72.78 eV in PtNi NPs and at 75.23 and 71.88 eV in PtPd NPs are attributed to the Pt^2+^ species. The binding energy of metallic-state Pt 4f_7/2_ in PdNi@PtNi NPs, PtNi NPs, and PtPd NPs exhibits a shift compared to the binding energy of pure Pt 4f_7/2_ (70.9 eV) [16,44]. This shift is attributed to the differences in electronegativity between Pt, Pd, and Ni, which generate more active sites, enhancing catalytic activity and weakening the binding strength between Pt and intermediate species. Additionally, as illustrated in Figure 2d, the Pd 3d peaks at 335.75 eV and 341.00 eV in the PdNi@PtNi alloy and at 335.71 eV and 340.96 eV in the PtPd alloy are attributed to the Pd^0^ species [14,45]. The Pd 3d peaks observed at 337.57 eV and 342.82 eV in PdNi@PtNi NPs and at 337.52 eV and 342.77 eV in PtPd NPs correspond to the Pd^2+^ species. Moreover, the Ni^0^ 2P_3/2_ can be observed in PdNi@PtNi and PtNi (Figure 2b). The deconvolution of the XPS peaks (Figure 2b–d) shows that the majority of Pt, Ni, and Pd in PdNi@PtNi NPs are in metallic states. Likewise, most of the Pt and Ni in PtNi NPs and Pt and Pd in PtPd NPs are also in metallic states, indicating the formation of alloys. The composition of PdNi@PtNi NPs was analyzed using ICP-OES and XPS, with the results summarized in Table S2. It is well known that XPS measures surface element concentrations, while ICP-OES measures bulk element concentrations. Notably, the surface content of Pt (24.4%, measured by XPS) is significantly higher than the bulk content (10.1%, measured by ICP-OES). In contrast, the Pd content decreases from 60.1% in the bulk to 54.5% at the surface, suggesting that Pd atoms are embedded beneath Pt atoms, consistent with the HRTEM observations. Based on the combined HRTEM and XRD results, it is concluded that the PdNi@PtNi, PtNi, and PtPd alloy catalysts were successfully synthesized. The PdNi@PtNi sample exhibits a core–shell structure, with PdNi forming the core and PtNi as the shell. Additionally, the XRD and XPS analyses suggest that the incorporation of Pd and Ni induces compressive strain and modifies the electronic structure of Pt, resulting in a reduction in the d-band center and a weakening of the binding strength with intermediate species. This optimized electronic structure enhances the electrocatalytic performance of the catalyst.

**Figure 3:**
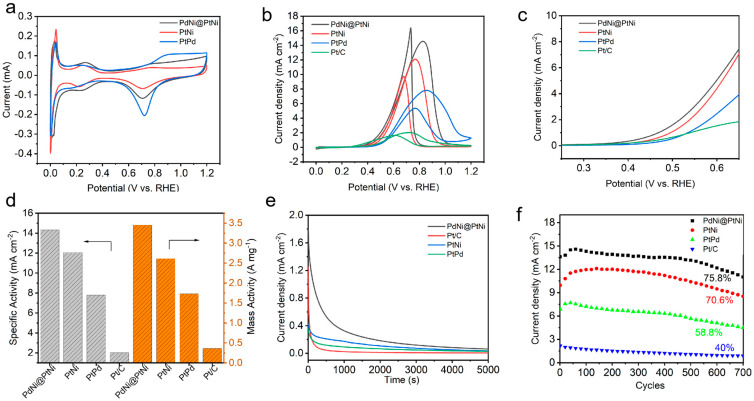
(**a**) CV curves for PdNi@PtNi NP, PtNi NP, and PtPd NP catalysts in a 1 M KOH solution at a scan rate of 50 mV⋅s^-1^. (**b**) Specific current densities of the catalysts; (**c**) onset potential of the catalysts; (**d**) specific activities and mass activities of the catalysts; (**e**) i-t curves of the catalysts; and (**f**) activity decay on different catalysts during the CV cycles.

The electrocatalytic activities of Pd@PtNi, PtNi, and PtPd catalysts were evaluated using EORs in a 1 M KOH solution with commercial Pt/C as a reference. Cyclic voltammetry (CV) was used to measure the electrochemical adsorption properties of the as-prepared PdNi@PtNi NPs, PtNi NPs, and PtPd NPs in the 1M KOH solution. Based on the CV curves, the electrochemical surface area (ECSA) of all catalysts was calculated by integrating the electric charges on the adsorption/desorption peak of the hydrogen regions. As illustrated in Figure 3a, the ECSA values for PdNi@PtNi NPs, PtNi NPs, PtPd NPs, and commercial Pt/C are 229 m^2^·gPt, 75.3 m^2^·gPt, 26.4 m^2^·gPt, and 45.7 m^2^·gPt, respectively (Table S3 and Figure S4). The ECSA represents the number of electrochemically active sites of Pt, reflecting the really active Pt sites in the surface of catalyst. It is noted that the ECSA of prepared catalysts are higher than that of commercial Pt/C. This can be explained by the small size of Pt alloy catalysts (<10 nm), which can increase the available active surface area, optimizing electronic properties by synergistic effects with alloying elements, thus improving the activity of the catalysts toward EORs.

The EOR performance of prepared PdNi@PtNi NP, PtNi NP, and PtPd NP catalysts and commercial Pt/C were conducted in a mixture of 1 M KOH and 1 M CH_3_CH_2_OH at a scan rate of 50 mV s^−1^. As shown in Figure 3b, the area-specific activity of PdNi@PtNi, PtNi, and PtPd catalysts was 14.31 mA·cm^−2^, 12.05 mA·cm^−2^, and 7.79 mA·cm^−2^, respectively, which is 7.05 times higher than that of commercial Pt/C (2.03 mA cm^−2^). From Figure 3c, PdNi@PtNi NPs exhibit an onset potential of 0.46 V (at a current density of 1.0 mAcm^2^) for the EOR, which is lower than that of PtNi NPs (0.49 V), PtPd NPs (0.53 V), and commercial Pt/C (0.54 V). As shown in Figure 3d and Figure S5, the max mass activity of ethanol oxidation on the catalysts is 3.35 A·mg^−2^ for PdNi@PtNi NPs, 2.81 A·mg^−1^ for PdNi@PtNi NPs, 1.73 A·mg^−2^ for PdNi@PtNi NPs, and 0.36 A·mg^−2^ for commercial Pt/C. The mass activity of PdNi@PtNi NPs is 9.30 times higher than that of commercial Pt/C, which shows remarkable activity among the reported electrocatalysts (Table S4). It is known that catalyst use in an alkaline medium is characterized by a higher Ni activity, so we also compared our results with Ni-based catalysts reported in the literature for ethanol electro-oxidation in alkaline environments [46,47,48]. As shown in Table S5, the PdNi@PtNi NPs exhibit higher mass activity than several Ni-based catalysts reported in the literature, including PdPtNi NPs (1.50 A·mg^−2^) [49], NiO@C/CC (1.99 A·mg^−2^) [50], and mPdNi/Ni NTs (1.52 A·mg^−2^) [51]. Previous DFT calculations showed that the Pd core in the PdNi@PtNi core–shell structure can modulate the electronic structure of the Pt shell, thus weaking the binding of OH_ads_ on the surface of the catalyst [52]. It is known that the oxidation removal of CO-based intermediates (CH_3_CO_ads_ and CO_ads_) by adsorbed OH (OH_ads_) is of critical importance in promoting electrochemical performance [11]. Therefore, modulating the electronic structure of the Pt shell can effectively accelerate the EOR kinetics. At the same time, at a sub-10 nm scale, the surface Pt atoms are more dispersed in the core–shell structure, which contributes to a more effective atom utilization [52].

The electrocatalytic stability of the PdNi@PtNi NPs, PtNi NPs, PtPd NPs, and commercial Pt/C were investigated by i-t curves at a potential of 0.4 V vs. RHE for 5000 s. As shown in Figure 3e, the current densities of PdNi@PtNi NP, PtNi NP, and PtPd NP catalysts far exceed that of commercial Pt/C. Moreover, the PdNi@PtNi NPs maintain the highest current density after the 5000 s test, indicating that PdNi@PtNi NPs show the best stability. To further test the catalytic stability of the as-prepared catalysts, we conducted multiple CV tests (700 cycles) for EORs. From Figure 3f, after 700 cycles, the activities of PdNi@PtNi NPs, PtNi NPs, and PtPd NPs remain at 75.8%, 70.6%, and 58.8% of the initial activities, respectively, while the commercial Pt/C only maintains 40.0%, further confirming the robust stability of the PdNi@PtNi NPs. Additionally, TEM analysis was also performed on the as-synthesized PdNi@PtNi NPs following electrochemical stability tests to assess the stability of catalysts. The TEM results (Figure S6) indicate that the structure of the PdNi@PtNi NPs remains well preserved after the durability tests. Compared to binary PtNi NP and PtPd NP catalysts, the higher stability of PdNi@PtNi NP catalysts may be attributed to the synergistic effects of the ternary alloys and its unique core–shell structure. All the data above show that the as-prepared ultrafine PdNi@PtNi NPs have excellent activity and stability compared to commercial Pt/C.

**Figure 4:**
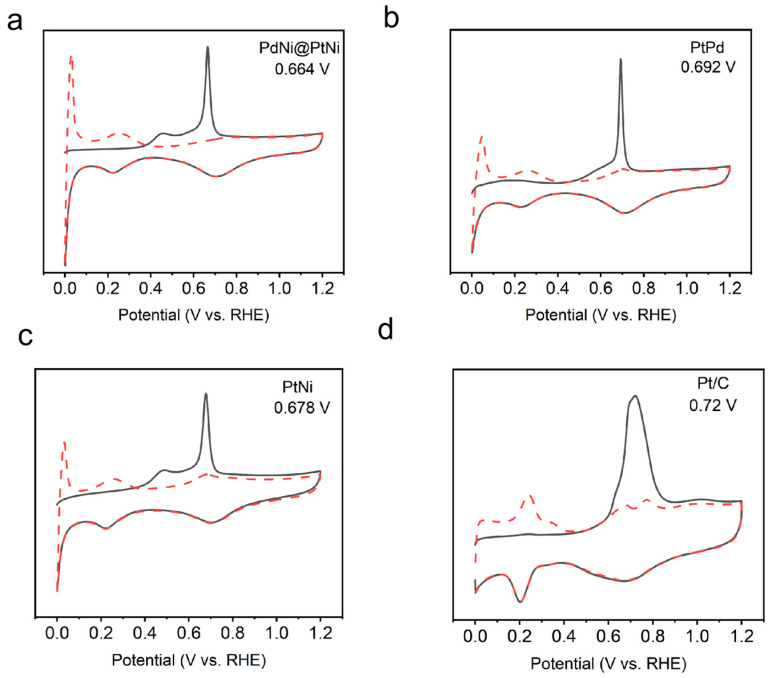
CO stripping test of (**a**) PdNi@PtNi NPs, (**b**) PtPd NPs, (**c**) PtNi NPs, and (**d**) Pt/C.

In alkaline EOR electrocatalysis, the oxidation of CO-based intermediates (CH_3_CO_ads_ and CO_ads_) by adsorbed OH (OH_ads_) is crucial for enhancing electrochemical performance. To simulate this oxidation process, we conducted an electrochemical CO stripping experiment. The CO stripping curves (Figure 4) show that the peak potential in the first cycle corresponds to the CO oxidation process. The peak potentials are 0.66 V for PdNi@PtNi NPs, 0.68 V for PtNi NPs, 0.69 V for PtPd NPs, and 0.72 V for commercial Pt/C. A lower peak potential indicates weaker CO adsorption on the catalyst surface, facilitating easier oxidation. Notably, PdNi@PtNi NPs, PtNi NPs, and PtPd NPs exhibit lower peak potentials compared to commercial Pt/C, likely due to the synergistic effects within the alloy structures that promote electronic charge transfer between the metals. Furthermore, the peak potential for CO oxidation on PdNi@PtNi NPs (0.66 V) is lower than that of PtNi NPs (0.68 V), PtPd NPs (0.69 V), and commercial Pt/C (0.72 V), suggesting that CO is more easily oxidized on PdNi@PtNi NPs. This implies that the unique core–shell structure of PdNi@PtNi weakens CO adsorption on the Pt surface, with the weaker Pt-CO bonding helping to mitigate CO poisoning, thereby enhancing the electrocatalytic performance for alcohol oxidation.

**Figure 5:**
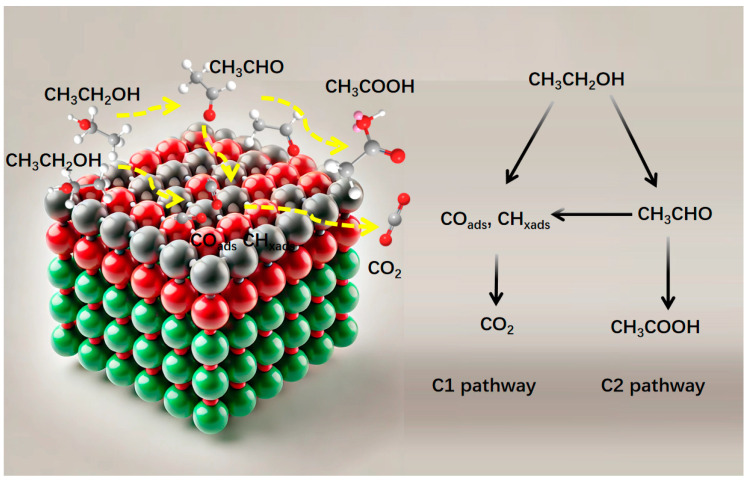
Reaction pathways for EORs at the Pt active sites.

Previous reports have shown that the electro-oxidation pathway of ethanol on Pt abides by two parallel but competing electrochemical pathways [11] (Figure 5). Typically, ethanol undergoes partial electro-oxidation via the C2 pathway, forming acetic acid through a four-electron exchange process (CH_3_CH_2_OH + 4OH^−^ → CH_3_COOH + 3H_2_O + 4 e^−^). In contrast, the C1 pathway involves the cleavage of the high-energy C─C bond, leading to the complete electro-oxidation of ethanol into more desirable products in an alkaline solution (CH_3_CH_2_OH + 16OH^−^ → 2CO_3_^2−^ + 11H_2_O + 12e^−^). The C1 process transfers 12 electrons and releases greater electrical energy, which is more electrochemically favorable for DEFCs. On the basis of the above results, we proposed a possible mechanism of how the PdNi@PtNi NPs promoted the high activity for EORs in an alkaline condition. On the one hand, the sub-10 nm metal core–shell structure produced more highly active sites and promoted the electron/mass transports, thus remarkably accelerating the EOR kinetics. On the other hand, compared to pure Pt, trimetallic PdNi@PtNi NPs are beneficial for electronic structure optimization. The synergistic effect between metals creates adjacent catalytic sites with differing properties, which can separate the chemisorption of ethanol’s carbon atoms and facilitate the C–C bond cleavage through adsorbed hydroxide (OH_ads_). The CO stripping results confirm that the synergistic effect plays a key role in enhancing anti-poisoning properties, resulting in increased catalytic activity. In addition, the interaction between the PtNi shell and the Pd core in PdNi@PtNi NPs is beneficial for the performance of EORs. The core–shell structure optimizes the binding energetics of intermediates on the Pt surface by adjusting the surface Pt electronic structure and strain. Therefore, the PdNi@PtNi NPs are highly efficient for new energy conversion, owing to their enhanced catalytic activity and stability.

## 3. Experimental Section

### 3.1. Synthesis of Catalyst

#### 3.1.1. Synthesis of PdNi@PtNi Core–Shell Nanoparticles

A total of 15 mg of palladium (II) acetylacetonate (Sigma-Aldrich, Saint Louis, MO, USA), 8 mg of platinum (II) acetylacetonate (Sigma-Aldrich, Saint Louis, MO, USA), 6 mg of nickel (II) acetylacetonate (Sigma-Aldrich, Saint Louis, MO, USA), 30 mg of ascorbic acid (AA, Sinopharm Chemical Reagent Co., Ltd. Shanghai, China), 20 mg of potassium bromide (KBr, Sinopharm Chemical Reagent Co., Ltd. Shanghai, China), and 50 mg of polyvinylpyrrolidone (PVP, Sinopharm Chemical Reagent Co., Ltd. Shanghai, China) were added into a vial containing 10 mL of N,N-Dimethylformamide (DMF, Sinopharm Chemical Reagent Co., Ltd. Shanghai, China). Place the vial in an ultrasonic cleaner and sonicate for 30 min to ensure uniform mixing. After sonication, transfer the mixture to a 25 mL reaction vessel and place the vessel in an oven at 160 °C to react for 3 h. After cooling, collect the product by centrifugation (10,000 rpm, 20 min) and wash it three times with ethanol and water to obtain the final product. Finally, redisperse the product in ethanol for further characterization.

#### 3.1.2. Synthesis of PtNi Nanoparticles

A total of 8 mg of platinum (II) acetylacetonate, 6 mg of nickel (II) acetylacetonate, 30 mg of AA, 20 mg of KBr, and 50 mg of PVP were added into a vial containing 10 mL of DMF. Place the vial in an ultrasonic cleaner and sonicate for 30 min to ensure uniform mixing. After sonication, transfer the mixture to a 25 mL reaction vessel and place the vessel in an oven at 160 °C to react for 3 h. After cooling, collect the product by centrifugation (10,000 rpm, 20 min) and wash it three times with ethanol and water to obtain the final product. Finally, redisperse the product in ethanol for further characterization.

#### 3.1.3. Synthesis of PtPd Nanoparticles

A total of 15 mg of palladium (II) acetylacetonate, 8 mg of platinum (II) acetylacetonate, 30 mg of AA, 20 mg of KBr, and 50 mg of PVP were added into a vial containing 10 mL of DMF. Place the vial in an ultrasonic cleaner and sonicate for 30 min to ensure uniform mixing. After sonication, transfer the mixture to a 25 mL reaction vessel and place the vessel in an oven at 160 °C to react for 3 h. After cooling, collect the product by centrifugation (10,000 rpm, 20 min) and wash it three times with ethanol and water to obtain the final product. Finally, redisperse the product in ethanol for further characterization.

### 3.2. Characterization

The morphology, structure, and elemental distribution of PdNi@PtNi, PtNi, and PtPd alloys were observed using a Tecnai F20 (FEI Company, Hillsboro, OR, USA) transmission electron microscope (TEM) and a high-angle annular dark-field scanning transmission electron microscope (HAADF-STEM) equipped with X-ray energy dispersive spectroscopy (EDS). X-ray diffraction (XRD) characterization of the prepared PdNi@PtNi, PtNi, and PtPd alloys was performed using a Shimadzu XRD 6000 (Shimadzu Corporation, Kyoto, Japan) powder X-ray diffractometer. The content of Pt, Ni, and Pd was measured by inductively coupled plasma optical emission spectroscopy (ICP-OES). Additionally, the surface composition and chemical valence states of the elements in the PdNi@PtNi, PtNi, and PtPd alloys were analyzed using X-ray photoelectron spectroscopy (XPS) and surface valence band emission spectroscopy on a PHI-1600 ESCA spectrometer (PerkinElmer, Waltham, MA, USA).

### 3.3. Electrocatalytic Testing

The electrocatalytic tests were conducted using a conventional three-electrode system on a CHI760e electrochemical workstation. A saturated calomel electrode (SCE) was used as the reference electrode, a platinum wire served as the counter electrode, and a glassy carbon electrode (GCE) with a diameter of 3 mm was used as the working electrode. The GCE was polished with Al_2_O_3_ powder of 1.0 μm, 0.3 μm, and 0.05 μm particle sizes and then washed with deionized water and ethanol. A total of 6 μL of the catalyst suspension (containing 6 μg of metal) was dropped onto the surface of the GCE and dried under an infrared lamp. Finally, 2 μL of 0.05 wt% Nafion solution (diluted with ethanol) was dropped onto the surface of the working electrode.

The electrochemical active surface area (ECSA) of the catalyst was tested using a 1 M KOH solution as the electrolyte. Prior to the experiment, high-purity N_2_ was purged through the electrolyte for 10 min to remove oxygen. Cyclic voltammetry (CV) scans were then performed at a scan rate of 50 mV/s, with the scan range set between 0 and 1.2 V. The atmosphere above the solution was maintained under N_2_ throughout the experiment. The electro-oxidation tests for ethanol were conducted in an electrolyte of 1 M KOH + 1 M CH_3_CH_2_OH. Before the CV tests, high-purity N_2_ was purged through the electrolyte for 10 min to remove dissolved oxygen. The scan range was set between 0 and 1.2 V, with a scan rate of 50 mV/s. The current density was expressed as the current per unit of the catalyst’s electrochemical active surface area (cm^2^) on the working electrode. A stable CV curve was obtained after cycling the working electrode 50 times at a scan rate of 50 mV/s. The multiple CV cycles (700 cycles) toward EORs were carried in a freshly prepared N_2_-saturated 1 M KOH solution at a scan rate of 100 mV/s. Chronoamperometry tests for EORs were carried out in a 1 M KOH + 1 M CH_3_CH_2_OH solution at a constant potential of 0.4 V.

## 4. Conclusions

In conclusion, we have successfully demonstrated a straightforward and well-designed synthesis of sub-10 nm PdNi@PtNi NPs with a core–shell structure via a one-pot solvothermal method for EOR catalysis. Compared to PtNi NPs, PtPd NPs, and commercial Pt/C, the sub-10 nm PdNi@PtNi NPs provide more active sites and facilitate improved electron and mass transport. Due to the synergistic interaction between the PtNi shell and the PdNi core, the binding energetics of intermediates on the Pt surface are optimized, resulting in significantly enhanced electrocatalytic performance. Benefiting from these features, the PdNi@PtNi NPs achieve an ethanol electro-oxidation area-specific activity of 14.31 mA·cm^−2^ and a mass-specific activity of 3.35 A·mg^−1^, which are 7.05 and 9.30 times higher than those of Pt/C, respectively. Additionally, the PdNi@PtNi NPs exhibit excellent stability for EORs. This study highlights the precise structural engineering of high-performance metal nanocatalysts through meticulous synthetic design.

## Data Availability

The original contributions presented in the study are included in the article and Supplementary Material, further inquiries can be directed to the corresponding author.

## Supplementary Materials

The following supporting information can be downloaded at: https://www.mdpi.com/article/10.3390/molecules29204853/s1. Details of materials and methods, as well as additional TEM images (PtNi NPs and PtPd NPs catalysts), XPS spectra(PdNi@PtNi, PtNi and PtPd catalysts) and electrochemical data. This material is available free of charge via the Supplementary Materials. References [37,46,47,49,50,51,53,54,55,56,57,58,59,60,61,62,63,64,65,66,67,68,69,70,71] are cited in the Supplementary Materials.
