# Supplementary material for: Sub-10 nm PdNi@PtNi Core–Shell Nanoalloys for Efficient Ethanol Electro-Oxidation
Source: Molecules. 2024 Oct 13;29(20):4853. doi: 10.3390/molecules29204853 (PMC11510317; doi:10.3390/molecules29204853)
Supplement: Supplementary file 1 [file molecules-29-04853-s001.zip › molecules-3227492-supplementary.pdf]

Supporting Information for

Sub-10-nm PdNi@PtNi Core-Shell Nanoalloys for Efficient Ethanol Electrooxidation

SuQian and Lei Yu *

School of Chemistry & Chemical Engineering and Environmental Engineering, Weifang University, Weifang 261061, China

Chemicals. Platinum (II) acetylacetonate ($Pt(C_5H_7O_2)_2$, $Pt(acac)_2$), Palladium (II) acetylacetonate ($Pd(C_5H_7O_2)_2$, $Pd(acac)_2$), Nickel (II) acetylacetonate ($Ni(C_5H_7O_2)_2$, $Ni(acac)_2$) were all purchased from SigmaAldrich. Polyvinyl pyrrolidone（($C_6H_9NO)_n$, PVP）, Ascorbic acid ($C_3H_7NO_6$, AA), Potassium bromide, (KBr, >99%) and N,N-Dimethylformamide (C_3H_7NO, DMF) were purchased from Sinopharm Chemical Reagent Co. Ltd. (Shanghai, China). All chemicals were used as
they were received, and no further purification process was performed during the experiment. The commercial Pt/C catalysts (Vulcan XC-72R) used for comparison were purchased from the Sainergy company. The loading of Pt content was about 40 wt %. The water used in all experiments was ultrapure (18.2 MΩ·cm).

CO-stripping measurements

For CO stripping measurements, a monolayer of CO was adsorbed on the working electrode through holding it at 0.1 V vs. RHE in N_2-saturated 1 M KOH under a flow of CO. Then the dissolved CO in the solution was removed by bubbling N_2 for 15 min. Then, two CV curves were recorded between 0 V and 1.2 V vs. RHE in N_2-saturated 1.0 M KOH solution at a scan rate of 50 mV s^{-1}.

The calculation methods

The ECSA ($m^2 g_{Pt}^{-1}$) of the catalysts is estimated according to the equation ECSA = $Q_H/(210 \times W_{Pt})$, where W_{Pt} represents the Pt loading (μg cm^{-2}) on the electrode, Q_H is the total charge (μC) for hydrogen desorption, and 210 represents the charge (μC cm^{-2}_{Pt}) required to oxidize a monolayer of hydrogen on a clean Pt surface. The area-specific activity is calculated as the ratio of current to the calculation of the active surface area of Pt by the adsorption/desorption peak of the hydrogen region, while the mass-specific activity is the ratio of current to the mass of the added catalyst.

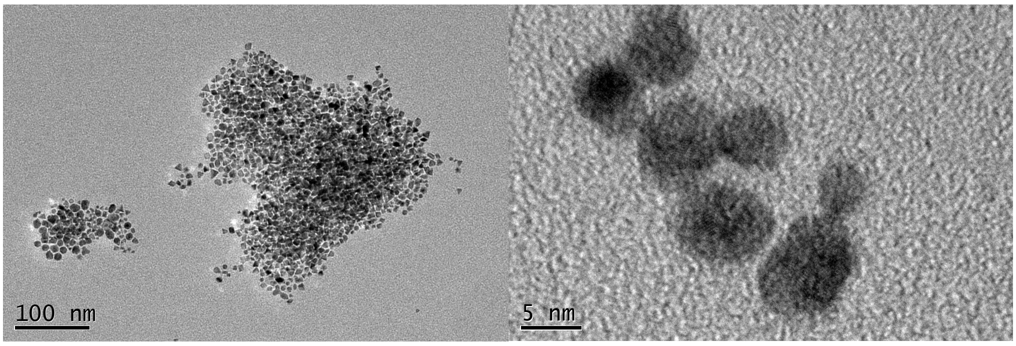

Figure S1. TEM images of PtNi NPs.

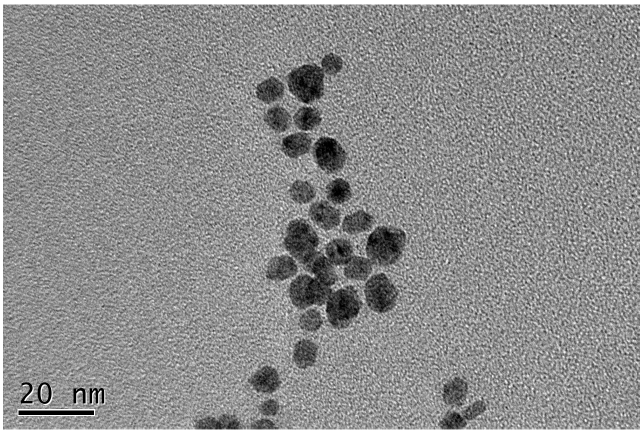

Figure S2. TEM image of PtPd NPs.

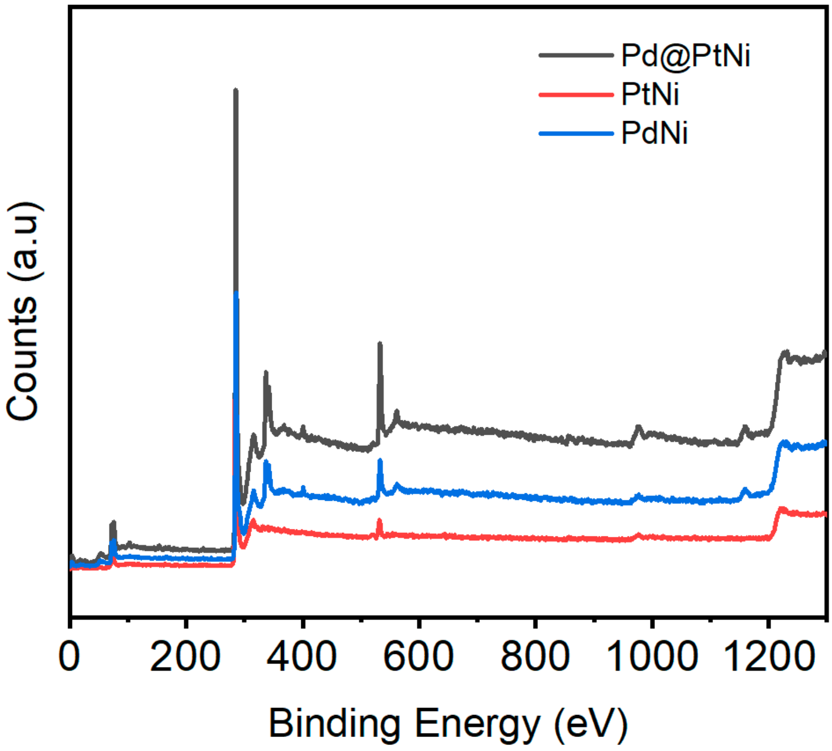

Figure S3. XPS spectra of PdNi@PtNi, PtNi and PtPd catalysts

Table S1. The contents of PdNi@PtNi NPs tested by ICP-OES

	Pt, %	Pd, %	Ni, %
PdNi@PtNi NPs	10.1	60.1	29.8
PtNi NPs	82.1	-	17.9
PtPd NPs	66.2-	33.7	

Table S2. The contents of PdNi@PtNi NPs tested by ICP-OES and XPS.

	Pt, %	Pd, %	Ni, %
ICP-OES	10.1	60.1	29.8
XPS	24.4	54.5	21.1

Table S3. The ECSA of PdNi@PtNi, PtNi and PtPd catalysts.

Catalysts	ECSA, m^2/g_{Pt}
PdNi@PtNi	229
PtPd	75.3
PtNi	26.4
Pt/C	45.7

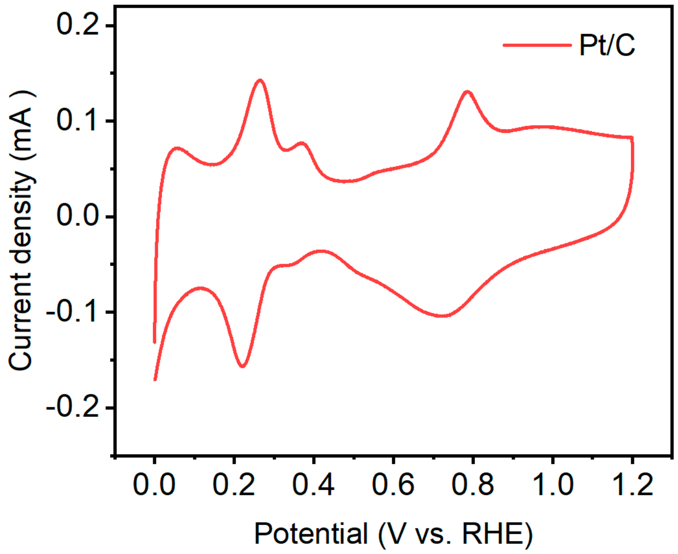

Figure S4. Cyclic voltammetry curves of Pt/C at a sweep rate of 50 mV s^{-1} in 1 M KOH.

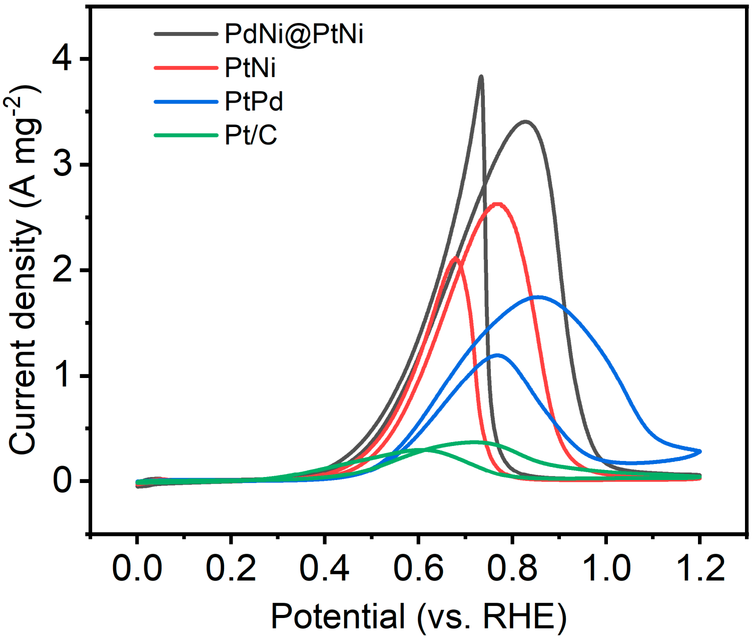

Figure S5. Mass current densities of PdNi@PtNi, PtNi and PtPd catalysts for EOR at a scanning speed of 50 mV/s.

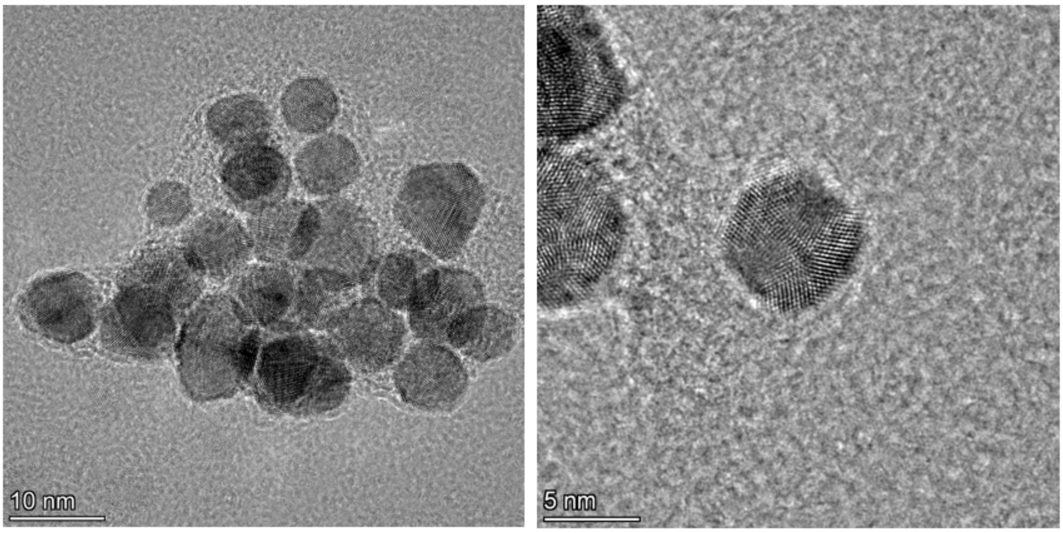

Figure S6. Mass current densities of PdNi@PtNi, PtNi and PtPd catalysts for EOR at a scanning speed of 50 mV/s.

Table S4. Electrocatalytic activities of prepared catalysts and other similar electrocatalysts for EOR in Alkaline medium from reported literatures.

Catalyst	Electrolyte	Specific Activity ($mA\ cm^{-2}$)	Mass activity ($mA\ mg^{-1}$)	Ref
**PdNi@PtNi NPs**	**1 M KOH + 1 M Ethanol**	**14.31**	**3350**	**This work**
**PtNi NPs**	**1 M KOH + 1 M Ethanol**	**12.05**	**2810**	**This work**
**PtPd NPs**	**1 M KOH + 1 M Ethanol**	**7.79**	**1730**	**This work**
Pd-Au HNS/C	1 M KOH + 1 M Ethanol	11.5	-	[1]
FFT Pt-Ir NRs	1 M KOH + 1 M Ethanol	10.22	-	[2]
PdCu SMPs	1 M KOH + 1 M Ethanol	-	6090	[3]
a-PdCu	1 M KOH + 1 M Ethanol	-	152500	[4]
Au@PtIr/C	1 M KOH + 1 M Ethanol	-	8300	[5]
PdAgSn/PtBi HEA NPs	1M KOH + 1M Ethanol	-	3386	[6]
PdCo NTAs/CFC	1M KOH + 1M Ethanol	-	1491	[7]
Pd/PANI/Pd SNTAs	1M NaOH + 1M Ethanol	-	~350	[8]
fcc Pd_3Sn	1M KOH + 1M Ethanol	-	2450	[9]
c-Pd-Ni-P@a-Pd-Ni-P	1M NaOH + 1M Ethanol	-	3050	[10]
PdZn NSs	1M NaOH + 1M Ethanol		2730	[11]
Pt/α-PtO_x/WO_3	0.1 M NaOH + 0.5 M Ethanol	-	2760	[12]
Pt1Mo1/C	0.5 M KOH + 1 M Ethanol	-	500	[13]
Pt-CeO_{2-x}/GNS	1 M KOH + 1 M Ethanol	-	1300	[14]

**Catalyst**	**Electrolyte**	**Specific Activity (mA cm^{-2})**	**Mass activity (A mg^{-1})**	**Ref**
**PdNi@PtNi NPs**	**1 M KOH + 1 M Ethanol**	**14.31**	**3.35**	**This work**
**PtNi NPs**	**1 M KOH + 1 M Ethanol**	**12.05**	**2.81**	**This work**
**PtPd NPs**	**1 M KOH + 1 M Ethanol**	**7.79**	**1.73**	**This work**
Pd/Ni(OH)$_2$@C/NF	1.0 M KOH+1.0 M Ethanol	-	1.3	[15]
mPdNi/Ni NTs	1.0 M KOH+1.0 M Ethanol	-	1.52	[16]
PdPtNi NPs	1.0 M KOH+1.0 M Ethanol	-	1.5	[17]
CuPdNiP NHs	1.0 M KOH+1.0 M Ethanol	-	1.19	[18]
NiO@C/CC	1.0 M KOH + 1.0 M Ethanol	119.1	1.985	[19]
Ni_xCo_{1-x} alloy	1.0 M KOH + 5.0 M Ethanol	142	-	[20]
Ni-B NTs	0.1 M NaOH + 0.5 M Ethanol	19.2	0.430	[21]
LDH@MnO$_2$	1.0 M KOH+1.0 M Ethanol	～4.5	-	[22]
NGr-NiO/Pulse	0.5 M NaOH + 1.0 M Ethanol	2.3	-	[23]
NiFe/ZrO$_2$/n-Si	1.0 M KOH+1.0 M Ethanol	34.4	-	[24]
Pd$_{80}$Ni$_{20}$/C	1 M NaOH + 0.5 M Ethanol	-	0.456	[25]

Table S5. Electrocatalytic activities of prepared catalysts and other Ni-based electrocatalysts for EOR in Alkaline medium from reported literatures.

References

1. Lv, F.; Zhang, W.; Sun, M.; Lin, F.; Wu, T.; Zhou, P.; Yang, W.; Gao, P.; Huang, B.; Guo, S. Au Clusters on Pd Nanosheets Selectively Switch the Pathway of Ethanol Electrooxidation: Amorphous/Crystalline Interface Matters. *Adv. Energy Mater.* **2021**, *11*. https://doi.org/10.1002/aenm.202100187.
2. Fang, Y.; Guo, S.; Cao, D.; Zhang, G.; Wang, Q.; Chen, Y.; Cui, P.; Cheng, S.; Zuo, W. Five-fold twinned Ir-alloyed Pt nanorods with high C1 pathway selectivity for ethanol electrooxidation. *Nano Res.* **2022**, *15*, 3933–3939. https://doi.org/10.1007/s12274-021-4062-z.
3. Lv, H.; Sun, L.; Wang, Y.; Liu, S.; Liu, B. Highly Curved, Quasi-Single-Crystalline Mesoporous Metal Nanoplates Promote C—C Bond Cleavage in Ethanol Oxidation Electrocatalysis. *Adv. Mater.* **2022**, *34*. https://doi.org/10.1002/adma.202203612.
4. Wang, W.; Shi, X.; He, T.; Zhang, Z.; Yang, X.; Guo, Y.-J.; Chong, B.; Zhang, W.-M.; Jin, M. Tailoring Amorphous PdCu Nanostructures for Efficient C–C Cleavage in Ethanol Electrooxidation. *Nano Lett.* **2022**, *22*, 7028–7033. https://doi.org/10.1021/acs.nanolett.2c01870.
5. Liang, Z.; Song, L.; Deng, S.; Zhu, Y.; Stavitski, E.; Adzic, R.R.; Chen, J.; Wang, J.X. Direct 12-Electron Oxidation of Ethanol on a Ternary Au(core)-PtIr(Shell) Electrocatalyst. *J. Am. Chem. Soc.* **2019**, *141*, 9629–9636. https://doi.org/10.1021/jacs.9b03474.
6. Lao, X.; Liao, X.; Chen, C.; Wang, J.; Yang, L.; Li, Z.; Ma, J.; Fu, A.; Gao, H.; Guo, P. Pd-Enriched-Core/Pt-Enriched-Shell High-Entropy Alloy with FaceCentred Cubic Structure for C1 and C2 Alcohol Oxidation. *Angew. Chem. Int. Ed.* **2023**, *62*, e202304510.
7. Wang, A.L.; He, X.J.; Lu, X.F.; Xu, H.; Tong, Y.X.; Li, G.R. Palladium–Cobalt Nanotube Arrays Supported on Carbon Fiber Cloth as High-Performance Flexible Electrocatalysts for Ethanol Oxidation. *Angew. Chem. Int. Ed.* **2015**, *54*, 3669–3673. https://doi.org/10.1002/anie.201410792.
8. Wang, A.-L.; Xu, H.; Feng, J.-X.; Ding, L.-X.; Tong, Y.-X.; Li, G.-R. Design of Pd/PANI/Pd Sandwich-Structured Nanotube Array Catalysts with Special Shape Effects and Synergistic Effects for Ethanol Electrooxidation. *J. Am. Chem. Soc.* **2013**, *135*, 10703–10709. https://doi.org/10.1021/ja403101r.
9. Zhou, M.; Liu, J.; Ling, C.; Ge, Y.; Chen, B.; Tan, C.; Fan, Z.; Huang, J.; Chen, J.; Liu, Z.; et al. Synthesis of Pd3Sn and PdCuSn Nanorods with L12 Phase for Highly Efficient Electrocatalytic Ethanol Oxidation. *Adv. Mater.* **2021**, *34*. https://doi.org/10.1002/adma.202106115.
10. Yin, P.F.; Zhou, M.; Chen, J.; Tan, C.; Liu, G.; Ma, Q.; Yun, Q.; Zhang, X.; Cheng, H.; Lu, Q.; et al. Synthesis of Palladium-Based Crystalline@Amorphous Core–Shell Nanoplates for Highly Efficient Ethanol Oxidation. *Adv. Mater.* **2020**, *32*. https://doi.org/10.1002/adma.202000482.
11. Yun, Q.; Lu, Q.; Li, C.; Chen, B.; Zhang, Q.; He, Q.; Hu, Z.; Zhang, Z.; Ge, Y.; Yang, N.; et al. Synthesis of PdM (M = Zn, Cd, ZnCd) Nanosheets with an Unconventional Face-Centered Tetragonal Phase as Highly Efficient Electrocatalysts for Ethanol Oxidation. *ACS Nano* **2019**, *13*, 14329–14336. https://doi.org/10.1021/acsnano.9b07775.
12. Xiao, L.; Li, G.; Yang, Z.; Chen, K.; Zhou, R.; Liao, H.; Xu, Q.; Xu, J. Engineering of Amorphous PtOx Interface on Pt/WO3 Nanosheets for Ethanol Oxidation Electrocatalysis. *Adv. Funct. Mater.* **2021**, *31*. https://doi.org/10.1002/adfm.202100982.
13. Pech-Rodríguez, W.J.; González-Quijano, D.; Vargas-Gutiérrez, G.; Morais, C.; Napporn, T.W.; Rodríguez-Varela, F.J. Electrochemical and in situ FTIR study of the ethanol oxidation reaction on PtMo/C nanomaterials in alkaline media. *Appl. Catal. B: Environ.* **2017**, *203*, 654–662. https://doi.org/10.1016/j.apcatb.2016.10.058.
14. He, Q.; Shen, Y.; Xiao, K.; Xi, J.; Qiu, X. Alcohol electro-oxidation on platinum–ceria/graphene nanosheet in alkaline solutions. *Int. J. Hydrogen Energy* **2016**, *41*, 20709–20719. https://doi.org/10.1016/j.ijhydene.2016.07.205.
15. Li, C.; Wen, H.; Tang, P.-P.; Wen, X.-P.; Wu, L.-S.; Dai, H.-B.; Wang, P. Effects of Ni(OH)2 Morphology on the Catalytic Performance of Pd/Ni(OH)2/Ni Foam Hybrid Catalyst toward Ethanol Electrooxidation. *ACS Appl. Energy Mater.* **2018**, *1*, 6040–6046. https://doi.org/10.1021/acsaem.8b01095.
16. Wang, H.; Jiao, S.; Liu, S.; Zhang, H.; Xu, Y.; Li, X.; Wang, Z.; Wang, L. PdNi/Ni Nanotubes Assembled by Mesoporous Nanoparticles for Efficient Alkaline Ethanol Oxidation Reaction. *Chem. —A Eur. J.* **2021**, *27*, 14472–14477.

17. Ren, G.; Liu, Y.; Wang, W.; Wang, M.; Zhang, Z.; Liang, Y.; Wu, S.; Shen, J. Facile Synthesis of Highly Active Three-Dimensional Urchin-like Pd@PtNi Nanostructures for Improved Methanol and Ethanol Electrochemical Oxidation. *ACS Appl. Nano Mater.* **2018**, *1*, 3226–3235. https://doi.org/10.1021/acsanm.8b00438.
18. Chen, D.; Zhang, R.-H.; Hu, Q.-Y.; Guo, Y.-F.; Zhan, W.; Chen, S.-N.; Zhou, X.-W.; Dai, Z.-X. Enhancing Ethanol Oxidation Reaction Activity of P-Doped CuPdNi Nanocatalyst by Optimizing Surface-Atom Distribution. *ACS Appl. Energy Mater.* **2019**, *2*, 5525–5533. https://doi.org/10.1021/acsaem.9b00715.
19. Liu, C.; Zhou, W.; Zhang, J.; Chen, Z.; Liu, S.; Zhang, Y.; Yang, J.; Xu, L.; Hu, W.; Chen, Y.; et al. Air-Assisted Transient Synthesis of Metastable Nickel Oxide Boosting Alkaline Fuel Oxidation Reaction. *Adv. Energy Mater.* **2020**, *10*. https://doi.org/10.1002/aenm.202001397.
20. Barakat, N.A.M.; Motlak, M.; Elzatahry, A.A.; Khalil, K.A.; Abdelghani, E.A.M. NixCo1−x alloy nanoparticle-doped carbon nanofibers as effective non-precious catalyst for ethanol oxidation. *Int. J. Hydrog. Energy* **2014**, *39*, 305–316. https://doi.org/10.1016/j.ijhydene.2013.10.061.
21. Muench, F.; Oezaslan, M.; Rauber, M.; Kaserer, S.; Fuchs, A.; Mankel, E.; Brötz, J.; Strasser, P.; Roth, C.; Ensinger, W. Electroless synthesis of nanostructured nickel and nickel–boron tubes and their performance as unsupported ethanol electrooxidation catalysts. *J. Power Sources* **2013**, *222*, 243–252. https://doi.org/10.1016/j.jpowsour.2012.08.067.
22. Jia, Z.; Wang, Y.; Qi, T. Hierarchical Ni–Fe layered double hydroxide/MnO2 sphere architecture as an efficient noble metal-free electrocatalyst for ethanol electro-oxidation in alkaline solution. *RSC Adv.* **2015**, *5*, 83314–83319. https://doi.org/10.1039/c5ra15718h.
23. Daryakenari, A.A.; Hosseini, D.; Mirfasih, M.H.; Apostoluk, A.; Müller, C.R.; Delaunay, J.-J. Formation of NiO nanoparticle-attached nanographitic flake layers deposited by pulsed electrophoretic deposition for ethanol electro-oxidation. *J. Alloys Compd.* **2017**, *698*, 571–576. https://doi.org/10.1016/j.jallcom.2016.12.136.
24. Cai, Q.; Hong, W.; Jian, C.; Li, J.; Liu, W. High-Performance Silicon Photoanode Using Nickel/Iron as Catalyst for Efficient Ethanol Oxidation Reaction. *ACS Sustain. Chem. Eng.* **2018**, *6*, 4231–4238. https://doi.org/10.1021/acssuschemeng.7b04661.
25. Roy Chowdhury, S.; Banik, M.S.; Mahajan, A.; Kumar Bhattacharya, S. Anode Catalytic Activity of Palladium-Nickel Alloy Nanoparticles for Ethanol Oxidation in Alkali. *ChemistrySelect* **2020**, *5*, 9848–9856. https://doi.org/10.1002/slct.202002382.

.
